# Supplementary material for: A comprehensive analysis of e-CAS cell line reveals they are mouse macrophages
Source: Sci Rep. 2018 May 29;8:8237. doi: 10.1038/s41598-018-26512-3 (PMC5974405; doi:10.1038/s41598-018-26512-3)
Supplement: Supplementary file 1 — Supplementary information [file 41598_2018_26512_MOESM1_ESM.pdf]

A comprehensive analysis of e-CAS cell line reveals they are mouse macrophages

Elizabeth Evans, Romain Paillot, María Rocío López-Álvarez*

[a]Animal Health Trust, Centre of Preventative Medicine, Immunology, Lanwades Park, Newmarket, Suffolk, CB8 7UU, UK.
[b]Department of Biology and Biochemistry, University of Bath

*Corresponding Author.
Mailing Address: Animal Health Trust, Centre of Preventative Medicine, Lanwades Park, Newmarket, Suffolk, CB8 7UU, United Kingdom.
Phone: +44 (0) 1638 751000 ext. 1271.
Fax: +44 (0) 1638 555634.
E-Mail: maria.lopez@aht.org.uk

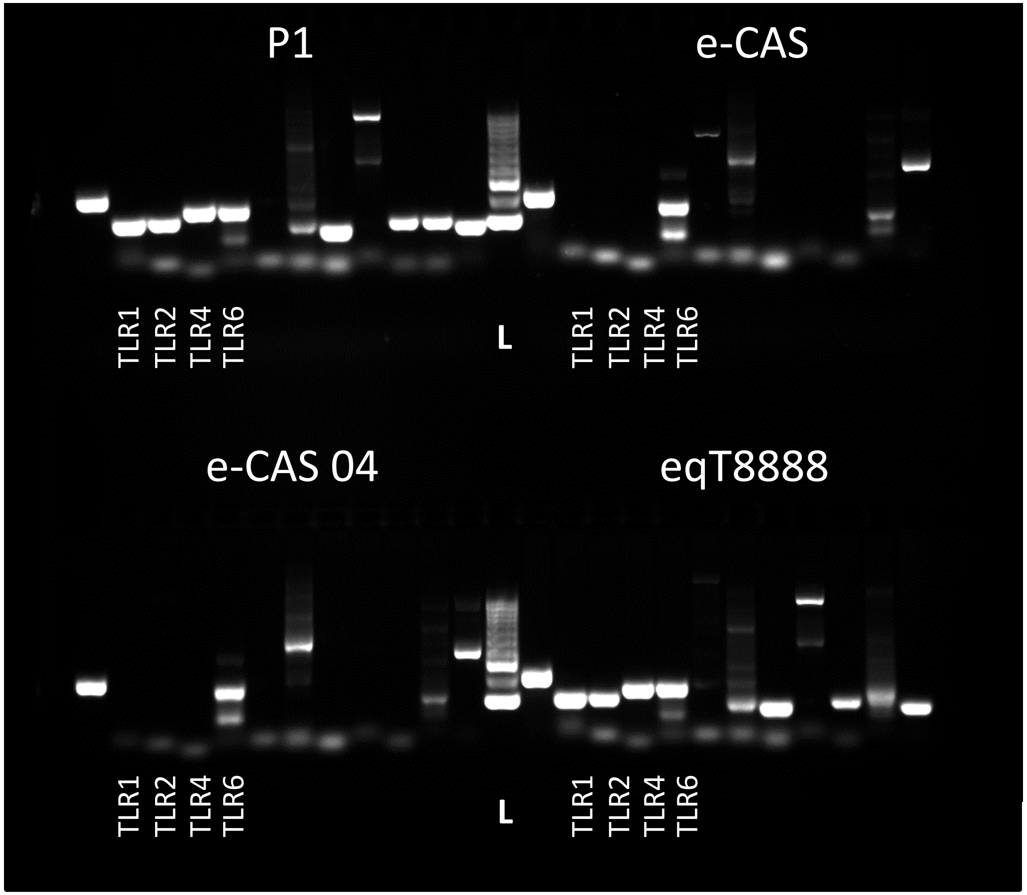

Supplementary Figure S1. *PCR reactions for equine TLRs (Original picture)* . Specific reactions for equine TLRs (lanes 2-5) performed with an archived DNA sample from a control pony (P1) and DNA samples obtained from two different aliquots of e-CAS cells (e-CAS and e-CAS 04) and from eqT8888 cells. L: Ladder. The rest of the reactions run in the same gel and shown in this image are not relevant for this article, although the difference between e-CAS and eqT8888 or P1 is quite clear. First lane shows the amplification of a reference gene (18s rRNA, Forward primer: ATGCGGCGGCGTTATTCC; reverse primer: GCTATCAATCTGTCAACTCCT)

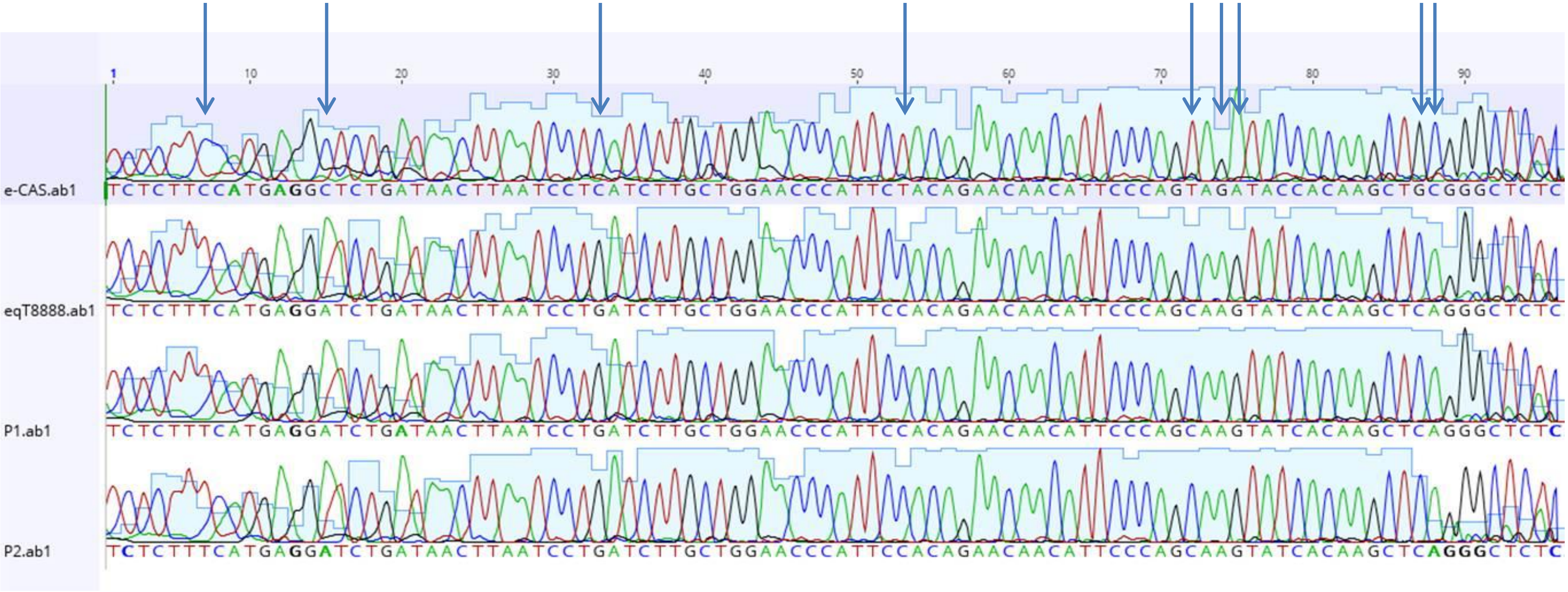

Supplementary Figure S2. *TLR6* aligned sequences from e-CAS, eqT8888, P1 and P2. Arrows indicate polymorphic sites

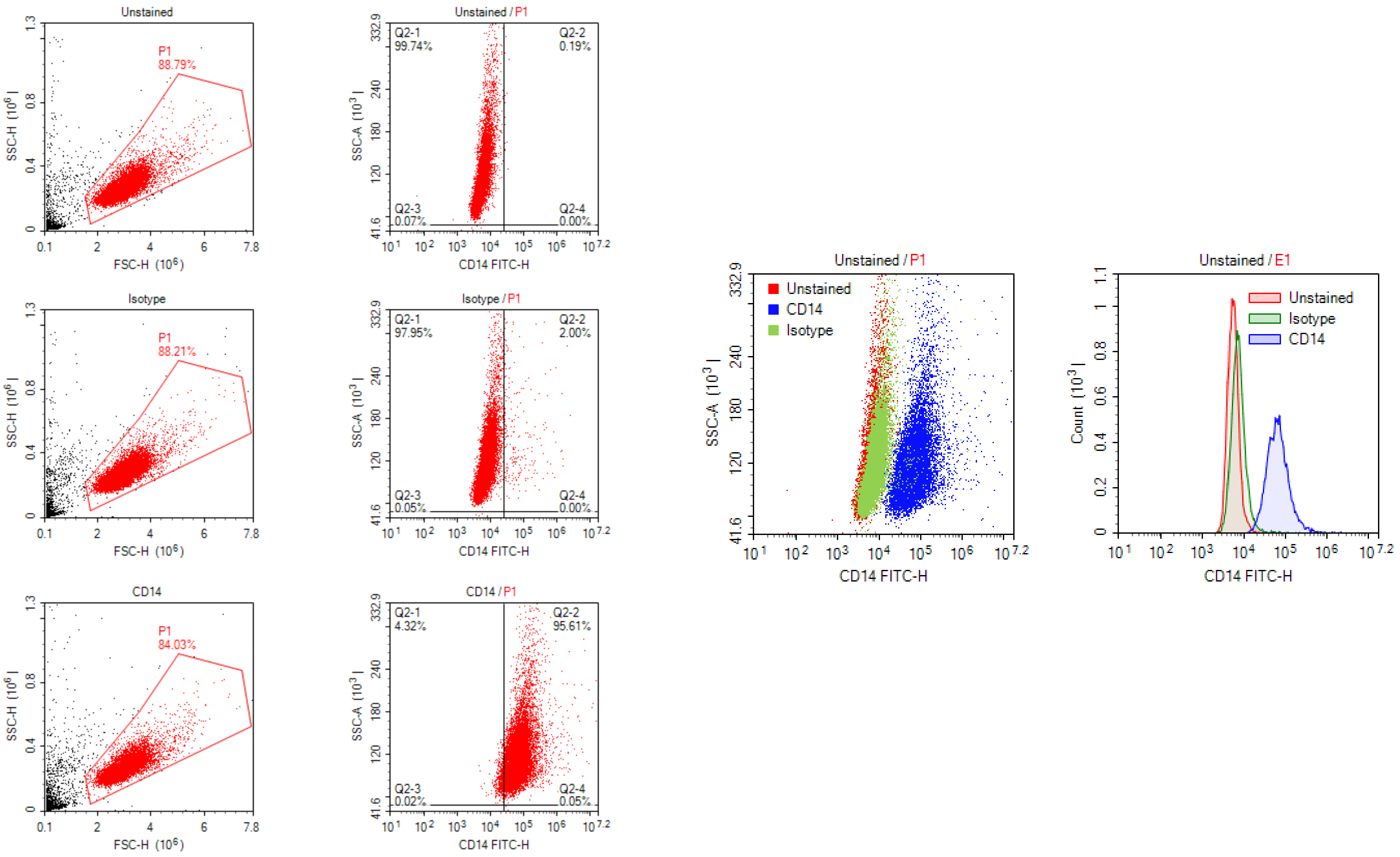

Supplementary Figure S3. *e-CAS cells are macrophages***.** Flow cytometry analysis showed that e-CAS cells were positive for CD14 (clone HCD14, FITC conjugated, Biolegend). FITC Mouse IgG1, κ Isotype Ctrl (clone MOPC-21, FITC conjugated, Biolegend) was used as isotype control. Samples were analysed using a NovoCyte Flow cytometer and the NovoExpress software (Acea Biosciences)
